# Innovation or deviation? The relationship between boundary crossing and audience evaluation in the music field

**DOI:** 10.1371/journal.pone.0203065

**Published:** 2018-10-18

**Authors:** Yongren Shi, Yisook Lim, Chan S. Suh

**Affiliations:** 1 Department of Sociology, University of Iowa, Iowa City, Iowa, United States of America; 2 Institute for Social Development Studies, Yonsei University, Seoul, Republic of Korea; 3 Department of Sociology, Chung-Ang University, Seoul, Republic of Korea; Northwestern University, UNITED STATES

## Abstract

Prior research in organizations has shown that the spanning of distinct social categories usually leads to an unfavorable reaction from the audience. In the music field, however, a recombination of categories has long been celebrated as a major source of innovation. In this research, we conduct a systematical research on the effect of spanning behavior by musicians with a particular focus on the structural heterogeneity of categorical boundaries. We first ask whether the blending of distinct music genres is penalized in the music field, and then investigate how the outcomes of spanning behavior are differentiated by the structural characteristics of each genres. After collecting a comprehensive dataset of musicians in the United States from diverse sources including AllMusic, iTunes, and MusicBrainz, we construct a two-mode network of musicians and subgenres. In calculating musicians’ genre-spanning behavior, we suggest a new diversity metric by incorporating the affinity between genres. Our results suggest that genre-generalist musicians who combine distinct music genres are more likely to be devaluated by listeners compared to genre-specialists who adhere to a single genre. Moreover, we find that musicians tend to be more penalized when they blend genres that have nonporous boundaries rather than penetrable boundaries. This research expands our understanding of the conditions under which boundary crossing leads to negative audience evaluation.

## Introduction

Taylor Swift, one of the most popular contemporary musicians in the United States, began her career as a traditional country singer-songwriter. Since the release of “The Story of Us” in 2010, however, she has combined her country tunes with pop music tunes in her music. Despite the initially mixed response from critics about her break from her country root, she eventually achieved great success in both country and pop music markets. Taylor Swift is not the only crossover artist who achieved fame and popularity from a broad array of audience by expanding the primary genre of music. Many successful musicians such as Michael Jackson, Miles Davis, and Prince, among others, are also well-known for their creative attempts to traverse multiple music genres throughout their career.

Despite these successful cases of genre spanning, we are still uncertain about whether these cases are generalizable to average musicians who build their career in the popular music field. On one hand, ordinary musicians who appeal to different types of listeners may receive a backlash by receiving negative connotations of being inauthentic and indistinguishable. Past literature in economic sociology and organizations has actually suggested that producers who cross the existing market categories are penalized for their lack of perceptual fit [1, 2]. Since existing categories structure the way in which the audience perceive products, producers who do not comply with the conventional classification system are likely to confuse both the critics and the general consumers. Empirical studies have consistently found a negative backlash towards producers who do not conform to market categories in various industries such as films [3], wines [4], software products [5], books [6], and restaurants [7].

Studies in the sociology of music have provided additional reasons to expect a negative effect of genre spanning on market performance. Each music genre forms a music community of musicians, critics, and listeners who exchange a certain manner of musical expression [8, 9, 10, 11]. Conventional classification of music genres not only influences the way musicians collaborate with others in the field of production, but also limits the type of music that potential consumers listen from the media. Moreover, some music genres are attached to strong group identities in terms of social class [9], race and ethnicity [12], or politics [13], making it hard for musicians to traverse the established genre boundaries [14]. Because of the significant influence of conventional music genres, we can expect that the audience in the music field may impose penalties to musicians who attempt to blend distinct music genres in their products.

On the other hand, however, cross-category innovation by musicians has consistently been a driving force in the development of music [11, 12, 15]; in case of other fields, see [16, 17, 18]. A mixing of distinct traditional genres has resulted in the creation of new music styles with new group identities over time. As the examples of blues rock, electronic rock, rap metal, and jazz hip-hop have shown, a fusion of traditional genres has been encoded into the finer classification system of subgenres. Accordingly, the music field is one of those artistic areas in which creativity and innovation are highly valued by the audience in the market. While musicians and recording companies are under the pressure to comply with the conventional classification of genres, critics and customers used to celebrate unique musicians who produce creative and eclectic music [19, 20]. Thus, it is probable that producers who span distinct music genres are not penalized but rather rewarded in the music field.

Building upon these conflicting expectations on category spanning in the music field, we test whether musicians’ spanning behavior of music genres influences their evaluation by the audience in the music field. Moving beyond the anecdotal stories of success and failure, we systematically examine whether musicians experience a backlash when they cross the boundaries of conventional genres. We explore our question under the premise that the outcome of spanning two genres will depend on the affinity between those genres within the larger classification system. Thus, we incorporate information on the conceptual distance among music genres in measuring the spanning of genres. Elaborating recent efforts to consider genre distance in measuring spanning behavior [6, 21], we suggest the Rao-Sterling index of diversity–a measure that gives more weights to an entity that combines distant categories rather than adjacent categories–to measure musicians’ propensity of spanning different genres.

Moving one step further, we investigate how the structural features of music genres matter in the relationship between category spanning and listeners’ evaluation. In particular, we examine whether the effect of spanning is contingent upon the boundary condition of the specific genres where they are embedded. Focusing on the network of musicians and their subgenres affiliations, we introduce the concept of *category porousness* to measure the strength of identities and norms within each genre communities. We test the possibility that musicians who span genres with porous boundaries are less likely to be penalized by listeners, while musicians who blend nonporous and insular genres are more likely to be devaluated by listeners.

To address these questions, we collect and combine a comprehensive data of musicians in the United States from diverse sources including Allmusic, Musicbrainz, and iTunes. The results from linear and multi-level regression analyses suggest that musicians who cross the boundaries of music genres over their career are associated with lower scores in their evaluation by listeners. Moreover, we find that this pattern is more salient for musicians who span music genres with nonporous boundaries than others who combine porous genres.

## Theoretical backgrounds

### Category spanning and market outcomes

Previous literature in organization theory has suggested that conventional classification system plays a critical role in shaping market outcomes [1]. With its distinct social categories, a classification system provides a conceptual tool for the audience to understand a variety of producers and their products in a market setting. When a producer or a product falls into a single social category, critics and consumers can easily make sense of the entity’s identity and, therefore, tend to evaluate the entity favorably. On the other hand, the audience finds it difficult to understand producers or products that simultaneously engage in multiple categories. Due to the confusing identity of the producers/products, the audience who internalizes the preexisting classification scheme is more likely to question the quality of the producers or products [3, 22].

Recent studies in category spanning have increasingly focused on how the structural characteristics of categories reshape the market outcome of spanning behavior [6, 23, 24, 25]. These studies have introduced the concept of *category contrast* to capture the distinctiveness/fuzziness of categorical boundaries. Social categories that entail stronger categorical codes and group identities are identified as having higher contrast in their boundaries than other categories [26, 27]. Research in this vein has argued that the level of penalty imposed on the spanning behavior of producers will be increased when the combined categories have high contrast to each other [24]. In other words, products that span two contrasting categories with crisp boundaries will confuse the audience even more. As a result, products will receive stronger negative reaction from the audience when they mix categories with clear boundaries rather than categories with fuzzy boundaries. Keuschnigg and Wimmer [25] further specified the conditions under which category-spanning behavior is penalized. In particular, they suggest that category-spanners are more likely to suffer when they combine culturally distant genres or when they are under a highly institutionalized market context.

Genres serve as the set of “categorical imperative” in the music field to provide listeners a perceptional framework to identify musicians and their products [8]. The classification system of genres reflects the shared understandings and expectations that listeners hold to musicians and their music [27]. Each music genre not only entails distinctive sounds and lyrics, but also provides symbols of membership for a particular social class or racial/ethnic group [8, 9, 10, 11]. For example, classical music has been strongly associated with a conservative, upper-class identity [9, 28], while hip-hop music has been initially associated with African Americans and their struggle [29]. Since the audience expects musicians to conform to the normative properties or social codes of genres, category-spanning musicians who do not fit with the genre-specific codes and features are perceived as being a non-member of any existing genre, and by extension, are avoided or discounted by the audience. Accordingly, the spanning of multiple genres can lead to a negative and unfavorable reaction by listeners. Following this categorical imperative mechanism, we hypothesize that *musicians who are listed in multiple genres/subgenres are more likely to receive lower ratings on their musical products than those who are listed in a single genre/subgenre*.

### Combinatorial innovation in music

Combinatorial innovation is defined as the collaborative process of connecting existing entities such as ideas, styles, designs, and others to generate a new entity [8, 16, 18, 30]. Combinatorial innovation is exemplified in the music field as the border-crossing activities of multiple preexisting genres. Border crossing has forcefully resulted in the creation of new genres and the subsequent development of the music scene [11, 12, 15].

While audience often expects musicians to comply with the conventional genre system, they also praise for musicians’ efforts to differentiate themselves from existing codes and to produce novel and innovative products [19, 20]. Accordingly, musicians who make attempts to fuse traditional music genres are respected as creative icons in the music field. A prominent example among jazz musicians is Miles Davis who radically integrated jazz tunes with other genres such as blues, rock, funk, and hip-hop throughout his career. Prince was also known as a musical innovator who created novel sounds by blending multiple music genres such as funk, rock, rhythm and blues, and pop. Due to these unique features of the music field, it is reasonable to expect that category spanners in the field of popular music are not as much penalized as they are in other industries.

Moreover, scholars have noted that the development of innovative online streaming technology over the past decades have greatly promoted cross-genre collaboration, and therefore blurred the boundaries between conventional genres [31, 32, 33]. Online music vendors such as the iTunes Music Store only provide simple and limited categorical information on musicians and their products; streaming service providers such as Last.fm and Pandora also do not offer any information regarding musicians’ genres. The weakening role of genre classification in online platforms may encourage listeners to navigate beyond established genre boundaries. Producers may also rely less on the preexisting classification system in their marketing strategies [33, 34]. Considering both the celebration of innovation and the weakening role of genre classification in the field of music, it is likely that the mechanism of categorical imperative is less significant in the music field. In other words, musicians who combine distinct genres/subgenres may not receive as much penalties as producers in other fields.

### Boundary porousness and audience evaluation

**Fig 1:**
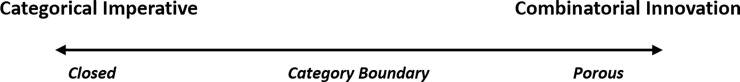
Boundary characteristics and audience evaluation.

Conflicting expectations exist over the effect of musicians’ genre spanning on their evaluation by audience. Herein, we explore the possibility that the strength of boundaries in music genres influences listeners’ evaluation. Inspired by the concept of category contrast [6, 23, 24], we introduce *category porousness* to measure the openness/closeness of the categorical boundaries of music genres. A genre is considered as having a low level of porousness when musicians tend to span subgenres within that major genre and do not cross the boundary of other major genres. Fig 1 illustrates the intervening role of boundary characteristics in the relationship between musicians’ spanning behavior and the listeners’ evaluation of it.

As the figure shows, we expect that the behavior of combining different music genres will be celebrated as combinatorial innovation if the boundary between the genres is porous. On the other hand, the combination of multiple genres with a nonporous boundary will tend to receive negative evaluation because the combined product will sound unusual and unfamiliar to the listeners.

On a cautionary note, we do not intend to establish a causal claim between the characteristics of categorical boundaries and the consequence of spanning behavior on ratings, as these two constructs reinforce each other in a self-perpetuating manner. When combinatorial innovation is celebrated in a certain cross-genre combination, musicians will more easily pursue novel music elements from other genres, forging an even stronger norm about music innovation. When a stringent norm of categorical imperative exist in a certain genre combination, on the other hand, musicians would less likely traverse the defined categorical boundary, producing an even stronger consensus of what constitute the “authentic” behavior that a musician should follow. Due to the nature of our cross-sectional data, we are unable to identify the causal direction between the categorical boundary characteristics and audience evaluation. Instead, we will focus on examining whether and how the audience’s reaction to category-spanning behavior varies by the porousness of categorical boundaries. Herein, we hypothesize that *musicians who are listed in multiple genres/subgenres are more likely to receive lower ratings on their musical products especially when they blend genres with nonporous boundaries*.

## Data and methods

### Music data collection

Our dataset was collected from three sources: MusicBrainz, AllMusic, and iTunes. We are interested in the field of popular music in the U.S. market; accordingly, we queried musicians’ geographical location information from MusicBrainz.com, a large-scale open-access database that is regularly maintained by users. MusicBrainz not only includes data on musicians’ personal information and recording histories, but also contains hyperlinks to other databases and websites. These hyperlinks could be used to check external validity from other sources with lower costs compared to conventional survey based research. An exhaustive SQL search yields 91,222 musicians whose geographical location tags includes states or territories of the United States.

After retrieving an exhaustive list of musicians from MusicBrainz.com, we obtained information on musicians’ genres from AllMusic.com. As the largest online music guide service, AllMusic.com provides a refined classification system of musicians by genres and subgenres. We follow the classification of AllMusic.com except for its integrated category of pop/rock. We believe that it is inaccurate to combine pop music and rock music given the distinct musical style and the identity of rock music [35]. Accordingly, we recode musicians’ genres to separate pop from rock based on the additional subgenre information provided by the AllMusic.com website. The genre/subgenre categories provided by allmusic.com are pulled for each individual artist from all of their albums. Our assessment of category-spanning behavior is on the basis of individual artist, rather than individual album. Using the Application Programming Interfaces (APIs) provided by AllMusic, we collected information on 11,857 musicians who engage in 22 primary genres and 329 subgenres.

Then, we collected customers’ average evaluation scores on musicians’ products from the iTunes Music Store, the largest commercial music downloading and sharing vendor in the U.S. We identified 10,596 musicians who have identifiable IDs in iTunes. Cross-referencing between MusicBrainz and iTunes is not a straightforward task due to the incomplete coverage of iTunes profiles in the MusicBrainz database. To address this issue, we first fetched musicians’ iTunes IDs by searching their names in the API, and then found customers’ ratings for each musicians with identifiable iTunes IDs. Given that customers’ ratings are given to albums rather than musicians, we assessed musicians’ appeal to their listeners by computing the weighted means of their albums’ ratings. The weights we used are the number of customers’ ratings that each album received. In total, we collected 8,029 musicians in 20 music genres who have rating information on iTunes. While iTunes is one of largest music vendors on the market, we couldn’t discern, and further analyze the data by, the demographics of the audience and the real motives behind the ratings.

We cannot totally rule out the possibility that there is a selection bias in collecting data from iTunes, especially given that we do not have full information on the demographic composition of iTunes users. At the same time, however, the direct ratings of the customers are a reliable source for assessing organizational performance especially when the website is used by a broad and comprehensive audience. Since iTunes is used by a comprehensive spectrum of customers due to its large market share, we are not seriously concerned about selection bias involved in collecting data from iTunes.

By combining data from Allmusic and iTunes, the level of analysis differs between category spanning (artist level) and audience evaluation (album level). This is not a serious limitation, however, since the genre/subgenre of artists is basically measured from the overall genre/subgenre of their albums in Allmusic. Thus, what we do with the data from iTunes–to compute the average evaluation of an artist from the evaluation of one’s albums–is similar to what Allmusic.com basically does. Since we are interested in the relationship between the cumulative spanning behavior of musicians and their cumulative evaluation, we are relying on the information from their overall productions. Table 1 shows an overview of the basic characteristics of primary genres in our dataset. We will use average individual ratings as the dependent variable in the analysis.

**Table 1:** Descriptive statistics of primary genres.

Primary Genres	Subgenre Count	Musician Count	Average Rating
Jazz	59	6,964	4.587
Blues	32	1,161	4.605
Rock	60	7,121	4.513
New Age	23	164	4.640
Electronic	38	1,236	4.415
Reggae	16	96	4.384
Rap	36	2,758	4.483
Latin	26	137	4.399
Country	27	1,009	4.496
Avant-Garde	20	183	4.437
Folk	19	367	4.557
Pop	43	1,774	4.413
R&B	16	2,078	4.456
Classical	16	2,045	4.511
Vocal	15	682	4.485
Easy Listening	18	180	4.541
Children’s	11	23	4.435
International	36	240	4.544
Holiday	6	32	4.634
Religious	3	273	4.611
Stage & Screen	3	309	4.409
Comedy/Spoken	2	163	4.441
Total	329	11,857	4.500

### Measuring category-spanning

Conventionally, the Simpson index of diversity [36] has been used to measure the extent to which an actor engages in a spanning behavior. Using this index, the degree of category spanning is measured as the number of categories that an actor combines. More categories an actor belongs to, the wider the actor spans across the conceptual space. If an actor is only listed in a single category, it means that the actor does not engage in a spanning behavior.

Despite its wide use in the previous research, the Simpson index is limited in that it does not consider the conceptual distance between the categories that an organization combines. Using this index, a combination of rock and blues is regarded equally as a mixing of hip-hop and country tunes.

Recent studies on categorization have developed alternative ways to incorporate conceptual distance in measuring the spanning of multiple categories [6, 21]. We extend these efforts by suggesting to use the Rao-Sterling index of diversity [37, 38, 39, 40] to measure categorical diversity. The Rao-Sterling index of diversity considers both the membership distribution of categories and the distances among categories. Our application of the Rao-Sterling index of diversity provides a rigorous representation of diversity since theindex can be reduced to the Simpson index of diversity when the distance is ignored.

The Rao-Sterling index considers three critical facets of diversity: variety, balance, and disparity [38]. *Variety* is the net number of categories that entities are assigned to. More categories an entity spans, the greater the diversity is. *Balance* captures the distribution in the strength of an entity’s membership in a category. When the assignment of an entity to a category is proportionally even across multiple categories, the assignment is balanced and the degree of diversity is maximal. In contrast, when a large fraction of the assignment is concentrated on a few categories, the assignment is unbalanced, yielding a low diversity level. Both variety and balance are captured in conventional diversity indices such as the Simpson Index in studies of ecology and the Herfindahl and Hirschman Index in economics. On the other hand, *Disparity* is a dimension that has mostly been neglected in the conventional application of diversity indices. Disparity refers to “the manner and degree in which elements may be distinguished” [38]. Two distinguishable categories are regarded as being distant to each other. Thus, an entity that is assigned to two distant categories is considered to have a higher level of diversity than an entity that spans two adjacent categories.

The Rao-Stirling index elegantly captures all three dimensions of diversity using the following formula:

$${width_{X}}^{Rao-Stirling}=\sum_{i\neq j}\theta_{Xi}*\theta_{Xj}*d_{ij}$$ (1)

where *d*_*ij*_ is the degree of disparity between categories *i* and *j*. In our case, it is measured as the distance between subgenres *i* and *j* on a two-mode network of entity and category. The distance between subgenres is measured based on how frequently they are connected by the same musician. For instance, rock & roll and contemporary blues are close to each other if they often co-occur in musicians’ profiles.

The concept of co-occurrence has increasingly been used as one of the critical dimensions in measuring the similarity between book genres, food cuisines [6], movies [7], and social entities on Twitter [41]. Considering that our dataset is not very large both in scale and in degree distribution, we use the Jaccard index as a way to capture co-occurrence between music subgenres. Jaccard index (*J*) is a function of the size of intersection divided by the size of union of two sets.

While distance and similarity are a pair of opposite poles, the exact formula for these two quantities is not well formed. We adopt the negative exponential function that has been used in measuring distances among categories [6, 7]:

Sim(i,j)=exp(−γd(i,j)) (2)

The above function is also known as Shepard’s law [42] which asserts that, with an empirical calibration, the perceived similarity between a pair of stimuli decays exponentially with distance. Then, the following equation provides us with the functional form of distance between categories *i* and *j*, with *γ* set to 0.25 [7]:

$$d(i,j)=-\frac{\ln\left(Jaccard(i,j)\right)}{\gamma }$$ (3)

The Rao-Stirling Index of Diversity is simply a generalized form of the Simpson Index, with distances between categories considered. If the distances between all pairs of categories are set to unity (*d*_*ij*_ = 1), then the Rao-Stirling index can be rewritten as:

$${width_{X}}^{Rao-Stirling}=\sum_{i\neq j}\theta_{Xi}*\theta_{Xj}*d_{ij}=\sum_{i\in L}\theta_{Xi}*\sum_{j\in L\&j\neq i}\theta_{Xj}$$ (4)

Herein, ∑_*j*∈*L*_*θ*_*Xj*_ = 1, and therefore, ∑_*j*∈*L*&*j*≠i_*θ*_*Xj*_+*θ*_*Xi*_ = 1. As a result, the rewritten form of the Rao-Stirling index is identical to the form of the Simpson index:

$${width_{X}}^{Rao-Stirling}=\sum_{i\in L}\theta_{Xi}*(1-\theta_{Xi})=\sum_{i\in L}\theta_{Xi}-\sum_{i\in L}{{(\theta }_{Xi})}^{2}=1-\sum_{i\in L}{{(\theta }_{Xi})}^{2}$$ (5)

It is noticeable that the formula of the Rao-Stirling index is identical to the total distance in Goldberg, Hannan, and Kovács [7] (Eq 2); the total distance is further used as an intermediary term for the calculation of object atypicality. Here, we highlight two methodological contributions. Firstly, we point out the connection of our revised index with the preexisting Simpson index used for niche width. We suggest that our measure can successfully replace the Simpson index if the information on the cognitive distance of categories is available. Secondly, our application of the Rao-Stirling index can contribute to organization studies that focus on diversity. We recommend the use of this index in measuring diversity on the basis of theorizing the concepts of variety, balance, and disparity of categories.

In computing the category-spanning of musicians, we rely on the information of subgenres rather than primary genres for two reasons. First, the classification of primary genres is too coarse-grained to be used for measuring category spanning. 11,857 musicians are assigned to 22 primary genres, with the assignment distribution being highly uneven. Pop music, the largest primary genre, includes close to 60 percent of all musicians (*N* = 6,964), while the two smallest primary genres, children’s music and holiday music, consist of only 0.2% (*N* = 23) and 0.3% (*N* = 32) of the musicians. Secondly, and more importantly, primary genres are unable to capture the constantly evolving and differentiating nature of the music scene. While primary genres have their own conventions, new subgenres are also likely to establish their own norms, and the consequences of the spanning of these distinct subcategories can only be observed using information on subgenres. Even if subgenres do not develop their clear musical boundaries, we can identify the significance of primary genres as an organizing principle by analyzing our data at the subgenre level. By using subgenre information, we are able to capture the influence of conventions and norms on musicians’ spanning activities at both genre and subgenre levels.

### Measuring Boundary porousness

Recent studies have found that the fuzziness/clarity of boundaries matters for organizations’ market outcomes. Boundary clarity has been identified as an important factor for audience to judge the quality and identity of producers in culture and product markets. The extent to which a category stands out has been measured by *category contrast*, the average typicality of members in a category [6, 23, 24].

Building on this effort spearheaded by Goldberg, Hannan, and Kovács [7], we extended the analysis to a more complex network system which comprises both musicians and subgenres. Using network analysis, our goal is to understand to what extent a primary music genre stands out from the rest of the music world in a network of interconnected musicians and their subgenres affiliations. We can find both the overall (central or peripheral) position of a genre with respect to every other genre and the connectedness among subgenres within the boundary of a primary genre.

Inside this network, we define boundary porousness as the extent to which a subgenre maintains network ties to the outside. Network ties indicate the co-listing of a musician to subgenres. To compute boundary porousness, we apply standard modularity index to each genre. In general, modularity is a measure that finds how closely one set of nodes are connected to each other compared to the complementary sets of all other nodes in the network [43].

Our network modularity index is appropriate to measure the characteristics of genre boundaries in the music field where primary genres and subgenres coexist in the genre classification system.

In mathematical form, the modularity of the weighted network can be formulated as:

$$Q=\frac{1}{2w}\sum_{i}\sum_{j}\left(w_{ij}-\frac{w_{i}w_{j}}{2w})\delta (C_{i},C_{j}\right)$$ (6)

where the edge weight *w*_*ij*_ is the Jaccard similarity between two subgenres *i* and *j*, 2*w* is the sum of all edge weights in the graph, 2*w* = ∑_*ij*_*w*_*ij*_, and *w*_*i*_ is the sum of all edge weights attached to subgenre *i*, *w*_*i*_ = ∑_*j*_*w*_*ij*_. *C*_*i*_ indicates node *i*’s community membership [43, 44, 45]. Modularity index *Q* compares the distribution of edges within and across boundary in the observed network with that in a randomized network. When *Q* is larger than zero, ties are more likely to occur between nodes sharing the same community membership, and it falls below zero when cross-boundary ties are more likely to form.

By comparing the connection pattern of the actual network with a randomized network, the index yields a neutral value of zero when the observed and randomized networks are undifferentiated to each other. The index yields a positive value when ties are more likely to be formed among members within a community compared to members outside the community. In our study, we refer low porousness to boundaries that are frequently traversed. Applying the concept of network modularity, we can investigate the relationship between the porousness of primary genres and the penalties imposed on musicians who cross over the subgenres within the boundaries of primary genres.

### Analytical strategy

We conduct a three-step analysis to examine the relationship between musicians’ category-spanning and listeners’ evaluation. In the first step, we map the network structure of music genres and subgenres using the classification information of musicians from Allmusic.com. This analysis shows clusters of subgenres and the relations between genres based on the frequency of co-listing by musicians. More frequently two subgenres are combined by musicians, closer the two subgenres are to each other; these subgenres are also more likely to be in the same cluster of the subgenre network. Based on the structure of subgenre networks, we can identify the clusters in the music scene that are not pre-defined. In other words, we can discover patterns of associations that are not recognized by experts.

In the second step, we shift the focus to musicians by exploring the association between musicians’ genre-spanning and their appeals to listeners. More specifically, we examine the relationship between the spanning behavior of musicians (*i*.*e*., musicians’ engagement in a diverse array of music subgenres) and the average rating they received from listeners in iTune over their careers. As noted in the previous section, we use the Rao-Stirling index of diversity to measure the spanning of musicians [37, 38, 39]. We employ an Ordinary Linear Squares (OLS) regression model to examine the relationship between category spanning and audience evaluation.

In the last step, we further explore whether the relationship between musicians’ genre-spanning and listeners’ evaluation is differentiated with the characteristics of primary genres. We apply linear hierarchical models to examine whether the relationship between the category spanning of musicians and their audience evaluation is moderated by the porousness of category boundaries at the primary genre level. Thus, primary genres are used as the upper-level groups under which distinct behavioral expectations are held for musician within those genres. On level-1 of the multi-level analysis (Eq 7), the rating of each musician is modeled based on the musician’s diversity (the Rao-Stirling diversity index at the subgenre level), the type of musicians such as forming a group, an orchestra, a choir and being a fictional character, and the total number of ratings registered on allmusic.com. We were unable to include musicians’ socio-demographic characteristics such as gender and career length since the sample size dramatically decreases if they are included. Applying listwise deletion to missing observations, the level-2 units include 20 music genres, corresponding to the groups (primary genres) that nest musicians.

We expect that average ratings of musicians vary across genres, and that the effect of subgenre diversity on audience evaluation is related to the relative strength of normative expectations (i.e., categorical imperative vs. combinatorial innovation) within each genre. In the first level-2 model (Eq 8), the intercept *β*_0*j*_ (a random effect) is modeled as a function of the mean of ratings of all genres (*γ*_00_) and a residual term that reflects difference of genre *j* from the overall mean (*μ*_0*j*_). In the second level-2 model (Eq 9), the slope *β*_1*j*_ (a random effect) for the predictor variable *diversity* is modeled as a function of the genre level variable *modularity*, measuring boundary porousness of genres. The interaction term (*γ*_11_) is built to test whether the effect of diversity on ratings is moderated by the porousness of the network boundary of genres. The subscript *i* indicates individual musicians, and *j* indicates primary genres. The statistical analysis is performed using R [46] and nlme [47].

Level-1:

$$Rating_{ij}=\beta_{0j}+\beta_{1j}(diversity)+\beta_{2}(type)+\beta_{3}(\log\left(\#Ratings\right))+r_{ij}$$ (7)

Level-2:

$$\beta_{0j}=\gamma_{00}+\mu_{0j}$$ (8)

$$\beta_{1j}=\gamma_{10}+\gamma_{11}(Modularity)+\mu_{1j}$$ (9)

Because the audience’s ratings on iTunes are right-censored at five stars, we conducted a robustness check using the same model in a Bayesian framework [48] using the R package brms (Bayesian multi-level models using Stan; [49]). The results from the Bayesian analysis are consistent with our main findings from the multi-level regression analysis.

## Results

### Mapping the genre and subgenre networks

**Fig 2:**
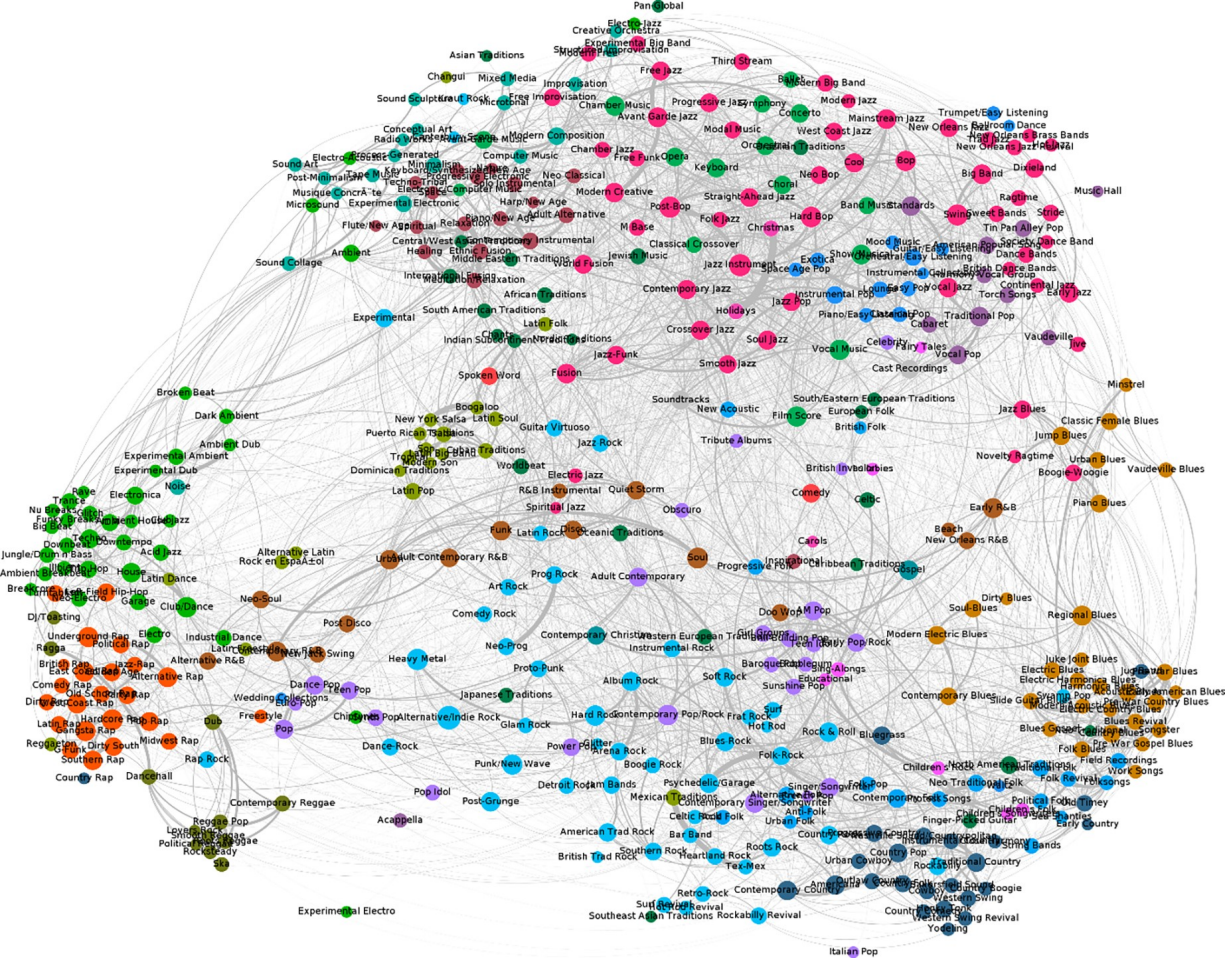
Network visualization of music genres and subgenres. *Note*: Nodes represent subgenres and edges represent the co-listing by the same musicians. More musicians shared by two subgenres, wider the edges are. Nodal colors indicate the nested primary genres.

Fig 2 shows the network structure of music subgenres based on co-listing of musicians to subgenres on Allmusic.com. Nodes represent subgenres and they are colored based on the primary genre in which they are nested. The edges that connect nodes are co-listing relationship and the width of the edges indicates the Jaccard similarity of co-listing musicians between subgenres. The layout algorithm generating this configuration is the force atlas 2 [50] which is based on the attraction and repulsion forces between nodes. More musicians share by two subgenres, the closer they are to each other in Fig 2.

The network visualization in Fig 2 displays a remarkable coincidence between the clusters emerging from the structure and the colors of nodes which are drawn from external categorization, indicating that subgenres from a same primary genre tend to be near one another. This finding confirms that primary genres serve as an organizing principle to bind its subgenres together–although the clusteredness of subgenres varies across primary genres. Subgenres of rap, electronic, reggae, and R&B music are clustered on the left, while subgenres of Latin music are in the middle. Subgenres of rock, pop, and country music are loosely centered at the bottom, and subgenres of Blues music are clustered at the bottom right. Additionally, subgenres of jazz, easy listening, vocal, folk, and new age are largely clustered on the top.

Upon an examination of the overall network visualization, we can identify three loosely connected music worlds. The first world is located on the bottom left consisting of genres such as rap, electronic, reggae, and R&B; the second is on the bottom right including pop, rock, country, and blues; the third world at the top includes primary genres such as jazz, classic, new age, and folk music. Our identification of these three music worlds fairly matches with the three clusters that Silver, Lee and Childress [34] found which are organized around hip-hop, rock & roll, and niche genres. Specifically, the first and second worlds of our network are similar to the two clusters found by Silver, Lee and Childress. The main genres of the third one are different, however, and we suspected that this difference is an outcome of the dissimilar data sources. Silver, Lee and Childress collected data from MySpace.com, an online social networking site frequented by high school youth, and the primary genres in our third cluster such as jazz, classic, new age, and folk music are likely to be underrepresented in their dataset.

All in all, the genre and subgenre networks demonstrate that the spanning of subgenres by musicians is structured by their primary genres. Our findings suggest that the particular conventions of primary music genres enable the spanning between their affiliated subgenres while constraining the connection to subgenres that are affiliated with other primary genres.

### Category spanning and listeners’ evaluation

Moving on to the main analysis in Table 2, we first employ regression models to further examine the relationship between musicians’ spanning behavior and their average rating from the audience. The OLS model in Model 1 reveals that there is a negative and statistically significant relationship between genre spanning and average ratings by listeners (−0.007, *p*<0.01). The results suggest that one unit increase in the diversity variable is related to 0.007 unit decrease in audience evaluation. To put the results in context, a one standard deviation increase in the diversity measure is associated with a decrease in the average musicians’ ratings by 0.015 stars in iTunes. While the effect is still considered to be weak, our result points to an overall pattern where musicians who span distant music subgenres are more likely to be devaluated by listeners in comparison to musicians who adhere to a single genre.

**Table 2:** OLS and Multi-level models on the relationship between category spanning and audience evaluation.

	OLS Regression	Multi-level Regression	
	Model 1	Model 2	Model 3
Diversity (category spanning)	−.007*** (.002)	−.006* (.004)	.005 (.005)
Modularity (boundary porousness)		.304 (.343)	.916** (.354)
Diversity * Modularity			−.157*** (.061)
Group Characteristics	.016* (.009)	.012 (.010)	.011 (.010)
Fictional Characteristics	−1.400*** (.268)	−1.359*** (.266)	−1.362*** (.266)
Orchestra	−.019 (.105)	−.023 (.105)	−.027 (.105)
Choir	.203 (.190)	.132 (.189)	.121 (.189)
Number of Rating	−.025*** (.002)	−.020*** (.002)	−.020*** (.002)
Constant	4.629*** (.013)	4.594*** (.029)	4.550*** (.029)
Random Effect:			
Residual		.375	.375
Intercept		.070	.051
Slope		.010	.007
Covariance		−.457	.171
R^2^	.022		
Log-Likelihood		−3,565.032	−3,564.393
Observation	8,029	8,029	8,029

Note

***p < 0.01

**p < 0.05

*p < 0.05 (one-tailed)

The correlation between genre spanning and listeners’ rating slightly decreases with our use of Rao-Stirling index in contrast to the preexisting Simpson index. Although the effect size slightly declines with the RS index, both of these correlations are statistically significant and in line with our theoretical expectation. Given that the correlation slightly decreases with the RS index, we believe that our new measure more accurately captures the penalties imposed to the spanning behavior of genres in the music field. In terms of model fit, the adjusted R-square of the OLS regression model using our Rao-Stirling index slightly increases in comparison to the regression model using the Simpson index. In other words, the fit between the data and the regression model improves with our use of the RS index.

Among control variables, forming a group is associated with an increase in one’s rating, while being a fictional character is negatively related to one’s evaluation by listeners. In addition, the volume of rating is negatively and significantly associated with a musician’s reception.

Next, we investigate the moderating effect of boundary porousness at the genre level using multi-level regression analysis. In Models 2 and 3, the Inter-Class Correlation (ICC) is 0.036, indicating that 3.6 percent of variance occurs across genres. The design effect of the model is 5.1. Both a non-zero ICC and a design effect greater than 2 imply the need for multi-level modeling [51, 52, 53].

The coefficient of the *diversity* variable in Model 2remains negative, but it loses some significance (−0.006, *p*<0.05 one-tailed). The main effect we look at here is the interaction between musicians’ *diversity* and the network *modularity* of music genres. Moving on to Model 3, we find a negative interaction effect (−0.157, *p*<0.01), which support our hypothesis on the moderating effect of boundary porousness in the spanning-evaluation relationship. Our results provide evidence that the effect of genre spanning on listeners’ evaluation is differentiated by porousness of music genres. Music genres with nonporous boundaries involve a stronger punishment on musicians who attempt cross-category innovation compared to genres with porous boundaries. A prominent example includes jazz music, the most nonporous genre with a modularity score of 0.18; musicians who cross the border of jazz are likely to be strongly penalized with an estimated coefficient of −0.023. On the other hand, the most porous genre, R&B, only involves an estimated effect −0.0014.

**Fig 3:**
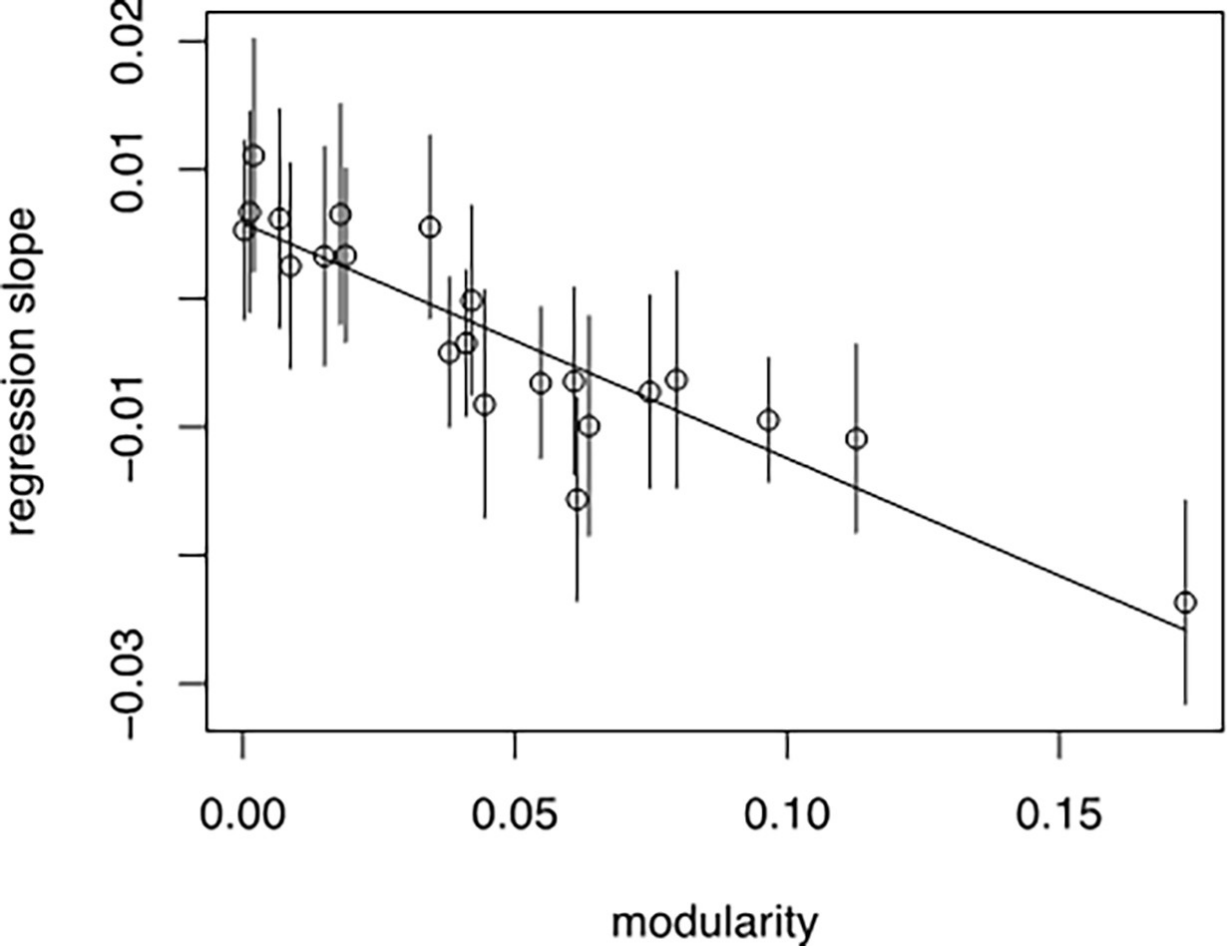
Boundary porousness and spanning-evaluation relationship.

We visualize our findings by plotting the slope of the second-level model in Fig 3.

In Fig 3, the dot and vertical lines are the estimates and standard errors for the genre slope *β*_1_, plotted versus genre-level network modularity, along with the estimated multi-level regression line *β*_1_ = *γ*_1_+*γ*_1_(*modularity*). The results show that the genre coefficients generally follow the regression line. The discrepancy of the coefficients from the line is summarized by the genre-level standard deviation parameter of 0.007. The figure illustrates that the negative relationship between genre spanning and audience evaluation is more salient in genres with low level of porousness.

## Discussion

Similar to other markets, conventional genre classification in the music field has a significant influence on the evaluation by critics and the listeners as well as the collaboration among musicians and the content distribution by media [8, 9, 10, 11]. At the same time, however, the music field is also an artistic area where innovative attempts by music artists to bridge different music genres can be highly valued by critics and the media [11, 12]. Our study is the first to examine whether cross-listing in multiple genres involves penalties in the music field. Specifically, we test whether musicians who cross the boundaries of music genres are likely to receive lower evaluation by listeners compared to those who stick with a single genre throughout their careers. In addition, we explore the possibility that penalties imposed on the spanning behavior of musicians are moderated by the porousness of categorical boundaries. Our findings suggest that genre-spanning musicians are more likely to receive lower rating on average compared to genre-specialist musicians in the market. Furthermore, the extent of penalty in spanning genres varies across different music genres. More penalties are imposed to musicians who cross over genres with nonporous boundaries.

Recent studies have focused on the heterogeneous effects of category spanning on the audience’s response across dissimilar categories. These studies have shown that categories having high contrast/low fuzziness are more likely to lose appeal to audiences than those with low clear boundaries [4, 6, 24]. Building upon these studies, the current paper introduces the concept of *boundary porousness* as an innovative network measure to show category identity and distinctiveness. By measuring the spanning of subgenres as the extent to which a subgenre maintains network ties to the outside, we capture in-group favoritism as well as out-group exclusivity. In-group favoritism may be the other side of out-group exclusivity and pertains to the strength of group norms and identity. Our findings suggest that the porousness of boundary in genres matters in shaping the evaluation of listeners on spanning activities. Music genres are likely to have nonporous boundaries if their music products are closely attached to particular values, styles, and identities of socio-economic or ethnic groups [9, 12, 14]. Accordingly, genre-spanning musicians are expected to receive more negative evaluation from the listeners when they combine genres with less porous boundaries. Our study provides an empirical support for this: our findings indicate that more penalties are imposed to the spanning of music genres that have nonporous boundaries. Due to negative reaction from the audience against the crossing of nonporous boundaries, conventional music genres with clear boundaries will be resilient to categorical innovation.

Our research also contributes to the methodology of studying the effect of category spanning. We incorporate the distance among music genres in computing the spanning of musicians. By using Rao-Sterling diversity index, we provide a more rigorous way to give weight to genre-spanners who combine distant genres. In addition, we use network modularity index to identify the varying characteristics of boundaries in each primary genres. In a field where primary and secondary genres exist within the multi-level classification system, modularity index may serve as an elaborated method to measure the porousness in genre boundaries.

It is likely that the negative effect of genre spanning is related to the expansion of the audiences as Kovác and Sharkey [54] indicated. We agree that popularity or market share of musicians can possibly mediate the relationship between boundary spanning and negative audience evaluation. When controlling for the number of ratings received by listeners as a proxy for popularity in our regression models, the main effect of spanning on evaluation does not change; but still, a more sophisticated time-series analysis may yield support for the mediating effect of popularity in the relationship between category spanning and audience evaluation.

While we use a large-N dataset to identify the broad pattern of spanning and its appeal to listeners, a data with more detailed information on the professional careers and socio-demographic characteristics of musicians will allow future studies to identify the causal relationship between genre spanning and audience evaluation. Moreover, a longitudinal data on musicians’ products will provide an opportunity to more rigorously examine the effect of genre-spanning activities on listeners’ rating in each stage of their careers [55]. Future studies can explore the possibility that the order which the musicians cross the boundaries matters or not in a longitudinal setting. Information on albums and songs, instead of musicians, will also give researchers a different angle to investigate the relationship between the spanning of genres and the evaluation of listeners. Despite these limitations of our research, our findings open the avenue for future research by identifying the relationship between category spanning and audience evaluation in the music field. This research provides implications to understand why conventional categories with crisp and nonporous boundaries are less likely to change over time.
